# “We didn't get much schooling because we were fishing all the time”: Potential impacts of irregular school attendance on the spread of epidemics

**DOI:** 10.1002/ajhb.23578

**Published:** 2021-02-17

**Authors:** Jessica Dimka, Lisa Sattenspiel

**Affiliations:** ^1^ Work Research Institute Oslo Metropolitan University Oslo Norway; ^2^ Department of Anthropology University of Missouri Columbia Missouri USA

## Abstract

**Objectives:**

Especially in traditional, rural, and low‐income areas, children attend school irregularly. School‐based interventions are common mitigation strategies for infectious disease epidemics, but if daily attendance is not the norm, the impact of schools on disease spread might be overestimated.

**Methods:**

We use an agent‐based model of an early 20th century Newfoundland community to compare epidemic size and duration in three scenarios: (1) all school‐aged children attend school each weekday, (2) students aged 10–15 have a chance of engaging in adult activities each day, and (3) students aged 10–15 have a chance of being reassigned to adult roles at the start of each simulation and thus never attend school.

**Results:**

As the probability of not attending school increases, epidemics become smaller and peak earlier. The change in final size is larger with permanent reassignment (35% at baseline, 18% at maximum reassignment) than with daily nonattendance (35% vs. 22%). For both scenarios, the peak occurs 3 days earlier with maximum absence compared to the baseline. Benefits extend beyond the reassigned agents, as all school‐aged agents are more likely to escape infection with increasing reassignment, and on average, 3–6 additional agents (2.6%–5.3%) escape infection compared to the baseline.

**Conclusions:**

This study demonstrates that absenteeism can have important impacts on epidemic outcomes. Thus, socioeconomic and other reasons for nonattendance of school, as well as how rates vary in different contexts, must be considered in models predicting epidemic outcomes or evaluating public health interventions in the face of major pandemics.

## 1 INTRODUCTION

The COVID‐19 pandemic has drawn attention to school‐based mitigation strategies against epidemics. Uncertainty about the role of and risk for schoolchildren has led to concern and debate over the costs, benefits, and safety of different approaches, especially closures or restricted attendance (e.g., Kroshus et al., 2020; Munro & Faust, 2020; Pierantoni et al., 2020; Sheikh et al., 2020). These public health interventions draw on evidence and assumptions that schoolchildren play significant roles in disease transmission throughout the community. Schoolchildren tend to have higher contact and attack rates than members of other age groups, lack prior immunity, become infected earlier during epidemics, and may have different symptoms, carry the virus for longer, or shed more of it (Fumanelli et al., 2016; Mossong et al., 2008; Munoz, 2002; Whitley & Monto, 2006). Despite these concerns, few epidemic models or analyses of data from past pandemics account for the range of variation in attendance due to reasons unrelated to health. Many children might attend school on an irregular basis or quit altogether before graduating. Without a full understanding of why and when children attend school, assuming regular attendance until graduation may overestimate the impact of this demographic group on disease spread.

Students fail to attend school for many reasons unrelated to health. For example, family responsibilities or economic concerns, such as housing instability, prevent regular attendance, while other students wish to avoid certain activities or interactions (e.g., bullying or harassment) (Balfanz & Byrnes, 2012; Cervantes, 2017). Absenteeism tends to be strongly associated with socioeconomic status, age, and gender (e.g., Havik et al., 2014; Schoeneberger, 2011). Another reason for absence from regular schools is homeschooling, particularly in the US, where one to two million children are homeschooled (Choi & Manning, 2010).

In 2015, 264 million children were not enrolled in school, accounting for 9% of primary school‐age students and up to 37% of upper secondary school‐age students. Seventeen million children, or 3% of the global population of primary school‐age children, will probably never enroll in school (UNESCO, 2017). Children in low‐ and middle‐income countries face significant barriers to education, which can lead to absenteeism and early dropout. Risks are associated with socioeconomic status, parental education, family size, sibling order, urban/rural location, disability, and gender (Bhattarai et al., 2020; Mizunoya et al., 2018; Wils et al., 2019). Absenteeism also may be due to significant infrastructure barriers, such as water, sanitation, and hygiene concerns (e.g., Dreibelbis et al., 2013; Kazeem et al., 2010; Kim & Rhee, 2019). Additionally, children in developing countries are often important sources of income or household production. Parents may believe it to be a better long‐term strategy to invest in the education of sons, who may be assumed to have higher earning potential and be more likely to provide support in the future compared to daughters (Kazeem et al., 2010).

Similar concerns also explain lower rates of attendance historically. Only 11% of children aged 14–17 years were enrolled in US high schools in 1900, increasing to more than 90% by 1978 (Grant & Eiden, 1980; Sherraden, 2008). Factors that appear to have influenced attendance include age, race, father's occupation, urban/rural location, and family structure (Denton & George, 1974; Moehling, 2004; Sherraden, 2008). Common reasons for girls dropping out were pregnancy and marriage, while economic reasons were reported for both boys and girls. Children contributed meaningfully to household production and agricultural labor, and during industrialization, children were in strong demand for factory work as well. Therefore, laws against child labor, welfare activism, and immigrant labor all contributed to shifts in attitudes and practices regarding school attendance over time (Sherraden, 2008).

The behaviors of school‐aged children may result in disease transmission to others at the household and community level, but previous studies suggest the role of schoolchildren in the wider epidemic spread is debatable. For example, France et al. (2010) calculated that only 79 of 702 household contacts of affected children became ill among a sample from New York City during the 2009 H1N1 pandemic. Some studies (e.g., Weycker et al., 2005) suggest that vaccination of schoolchildren can decrease the total number of influenza cases in a community. Yet, Talbot et al. (2009) analyzed rates of hospitalization for influenza from November 2006 to April 2007 in two Tennessee counties, one of which had a school‐based immunization program, and found no difference for individuals ≥50 years old. There were significantly lower rates for the 50–64‐year‐old age group, though, which the authors suggested could be due to the higher rates of vaccination among this age group in the intervention county rather than or in addition to the school program.

Nonetheless, illnesses among schoolchildren contribute to the significant social and economic costs of epidemics. Thorrington et al. (2017) found that children <15 years account for nearly 40% of all influenza‐related general practitioner consultations and hospital admissions in England. In one study of students in kindergarten through 8th grade in Seattle, for every 100 children affected, there were 63 school days missed and 20 days of work absences for parents (Neuzil et al., 2002). Yet, mitigation strategies like closures also can result in high costs, for example, due to parents staying home from work to care for children (e.g., Lempel et al., 2009).

Because of the potential social, economic, and health‐related costs associated with widespread epidemics that affect school‐aged children and their families, a substantial body of research has looked at the impact of planned or unplanned school closures on epidemic outcomes (e.g., Fumanelli et al., 2016; Jackson et al., 2013; Markel et al., 2007; Viner et al., 2020; Wu et al., 2010). However, the focus of this article is on scenarios and potential interventions when attendance patterns vary even when schools are open; thus, we do not focus on closures implemented only at the time of an epidemic. Much prior research, including that involving surveys and modeling studies, typically only considers absences due to illness from the disease being modeled or as a result of school closure (e.g., Barrett et al., 2011; Ciavarella et al., 2016; DePasse et al., 2017). Further, epidemiological studies often center on schools in high‐income countries or large cities (e.g., DePasse et al., 2017; Sugishita et al., 2019), which provides important insights into contact patterns and other features of contemporary schools, such as the structure of classrooms. For example, Cauchemez et al. (2011) found that the division of students into different grades and classes influenced transmission, while sex‐assortative playmates also led to different patterns and timing of spread among boys and girls.

Schools in many places around the world today and especially historically do not necessarily share this kind of structure, however. Schools might only have one or two rooms for students of all levels, be open only to one sex, or be long‐term boarding schools rather than day schools. Schools today with innovative curricula like service‐ or project‐based learning focused on independent work, internships, and/or community engagement would also lead to different rates of attendance and contact patterns among students, staff, and the larger population. To our knowledge, no studies have measured contact rates of school‐aged children who do not regularly attend school. Such research emphasizing standard styles of education in high‐income countries therefore insufficiently addresses potential impacts of nonattendance for different social and economic reasons.

Reduced attendance might alter the impact of schoolchildren on epidemic spread because of less mixing among students resulting in less transmission, or less involvement in school‐based public health interventions resulting in fewer vaccinated students or increased risk behaviors from a lack of health education. Irregular attendance also could create additional opportunities for contact between schoolchildren and other members of the community, facilitating varied routes of transmission. To better understand the patterns observed during historical epidemics, as well as the potential range of variation in outcomes today, it is necessary to consider behaviors among school‐aged children beyond health‐related absences and school closures. Communities where absences are likely to occur are also likely to suffer disproportionately during epidemics, due to a lack of resources and health services (e.g., Mamelund et al., 2013). Yet, such communities are rarely considered in epidemic simulation modeling compared to larger, more urban, or cosmopolitan settings.

In this article, we consider how the local context of a traditional Newfoundland community in the early 20th century could have influenced school attendance and, in turn, the spread of the 1918 influenza pandemic. We use an agent‐based simulation model to compare epidemic outcomes under different attendance scenarios. Agent‐based models are typically used to investigate dynamic processes across space and time, with a focus on system‐level properties that emerge from individual characteristics, behaviors, and interactions. In epidemic models, the size and duration of outbreaks are emergent outcomes of social contacts between individuals who are susceptible to the disease and those who are infectious. Agent‐based models explicitly consider individual variation, making them particularly suitable for studying small populations that are likely to be strongly influenced by heterogeneity as well as by random factors. In the following section, we describe the characteristics of the study population and how historical data are used to inform a model that allows us to explore how school attendance affects epidemic spread throughout the community.

## 2 MATERIALS AND METHODS

### 2.1 Study context and population

The 1918 influenza pandemic killed as many as 50–100 million people worldwide (Johnson & Mueller, 2002). On the island of Newfoundland, provincial death records show that there were almost 2000 deaths from influenza, pneumonia, and/or related causes. The average island‐wide mortality rate was about 74.5 per 10 000 people, although this varied widely by district (Sattenspiel, 2011).

**FIGURE 1:**
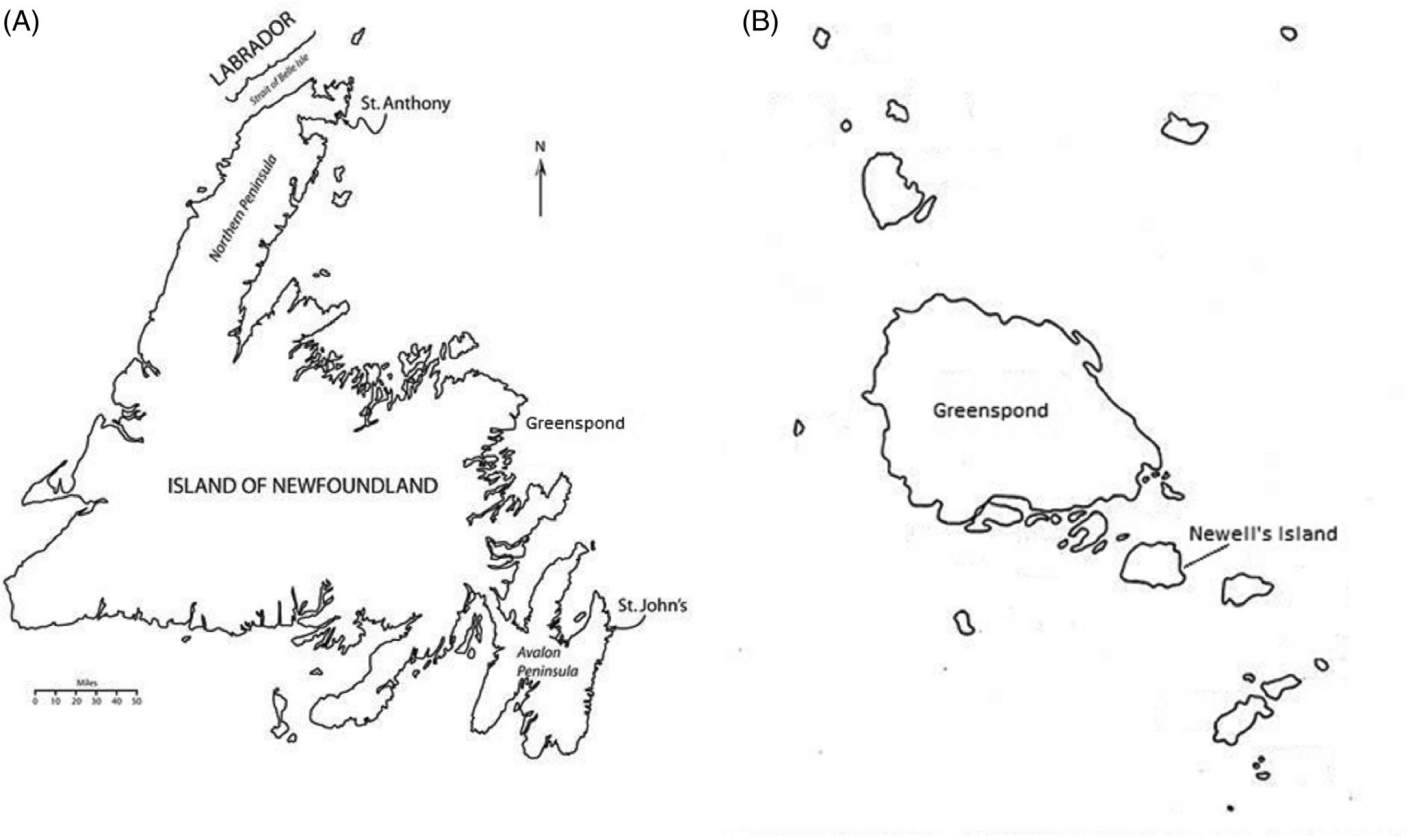
(A) Map of Newfoundland indicating the approximate location of Greenspond. Modified from Orbann et al. (2014), figure 6.1. (B) Greenspond and surrounding islands, including the study community of Newell's island. Modified from White (1994a)

Our model draws on demographic, archival, and ethnographic data for the study community of Newell's Island and other nearby regions. Now uninhabited, Newell's (in some sources, Newell) Island is part of a larger area comprising the community of Greenspond, which itself is an island off the coast of Newfoundland, and other small islands (Figure 1). The Greenspond region was strategically located for fishing, communication, and trade. The community included a resident doctor, magistrate, policeman, and customs officer, as well as a post office, courthouse, and several merchants and other businesses (Smallwood & Pitt, 1984; The Greenspond Come Home Year Committee, 1997). The population declined to an estimated 1500 by the time of the 1918 pandemic (Feltham, 1986; Winsor, 1979). Newell's Island lies ~230 meters to the southeast of the island of Greenspond (Feltham, 1986). Censuses from the 19th and 20th centuries indicate the population of Newell's Island fluctuated and was typically in the range of 70 to 100 people. The 1911 census, the last conducted before the 1918 pandemic, listed 114 residents (Colonial Secretary's Department, 1923; Newfoundland's Grand Banks, 2013; White, 1994c, 1999).

The October 29, 1918, issue of St. John's *Daily News* first mentioned the pandemic in Greenspond, reporting seven cases but no deaths. Weekly reports documented an increase in cases in late November through mid‐December. A telegram from the Greenspond magistrate dated December 9 reported 250 cases and “whole families…down with the disease, and…no assistance” (Provincial Archives of Newfoundland and Labrador, 1918). By December 17, the local doctor reported 400 cases to date and six deaths, but by December 24, the epidemic had subsided with no further deaths reported (The Influenza Epidemic, 1918a; The Influenza Epidemic, 1918b). Overall, 11 deaths are listed in mortality records for the Greenspond region (Provincial Archives of Newfoundland and Labrador, 1918‐1920). Using a population of 1500, this corresponds to ~73 deaths per 10 000, about the same as the average for Newfoundland as a whole. Morbidity was at least 27%, less than the estimated 50%–60% global morbidity (Ferguson et al., 2005; Morabia, 2020), although both estimates are uncertain given the difficulties inherent in inferring illness from historical records.

The exploitation of marine resources influenced the organizational patterns observed within Newfoundland communities, especially before Confederation with Canada in 1949. Households were patrilineal and patrilocal, with families commonly living in nuclear households. The fishing crews were typically formed of fathers, sons, and brothers (Davis, 1983; Firestone, 1967; Nemec, 1972; Queen & Habenstein, 1974). In addition to domestic work, many women participated in the fishery activities, performing virtually all of the tasks required by the shore crew (Murray, 1979; Porter, 1985; Prentice et al., 1988; Queen & Habenstein, 1974).

Generally, schools in Newfoundland were run by churches through the time of the 1918 pandemic (Winsor, 1979). These schools were typically one room, although, in the study region, two rooms were also common (Feltham, 1986; White, 1994b, 2002). Occasionally during the region's history, schools operated on the smaller islands as well as on Greenspond itself. A renovated store served as a school on Newell's Island in 1868, but this was subsequently closed around 1875 due to low enrollment. The school reopened ca. 1879–80 when the population increased again, and it continued operation until 1918 with enrollment consistently around 20 students (Winsor, 1979). There is no indication that this last closure was related to the pandemic. Homeschooling practices similar to those seen today do not appear to have been common. Although opinions vary in some sources (e.g., Feltham, 1987), education was highly valued in the region, perhaps due to the relatively large population size and community resources. However, as in other historical or rural populations, it was common for children to quit school or attend inconsistently as they grew older due to socioeconomic circumstances or seasonal activities (White, 1994b, 1998, 2000). Interviews published in the community's historical newsletter, *The Greenspond Letter*, illustrate this behavior. One interviewee born in 1900 said, “we didn't get much schooling because we were fishing all the time” (White, 1998), while another, born 1913, said, “I only had grade seven…I was the oldest, see, the only one to help” (White, 2000). The weather also could influence attendance by preventing travel to the school (White, 2003). This sort of variation in school attendance could therefore have played an important role in how infectious diseases, including the 1918 pandemic, spread through the community.

### 2.2 Model description

**FIGURE 2:**
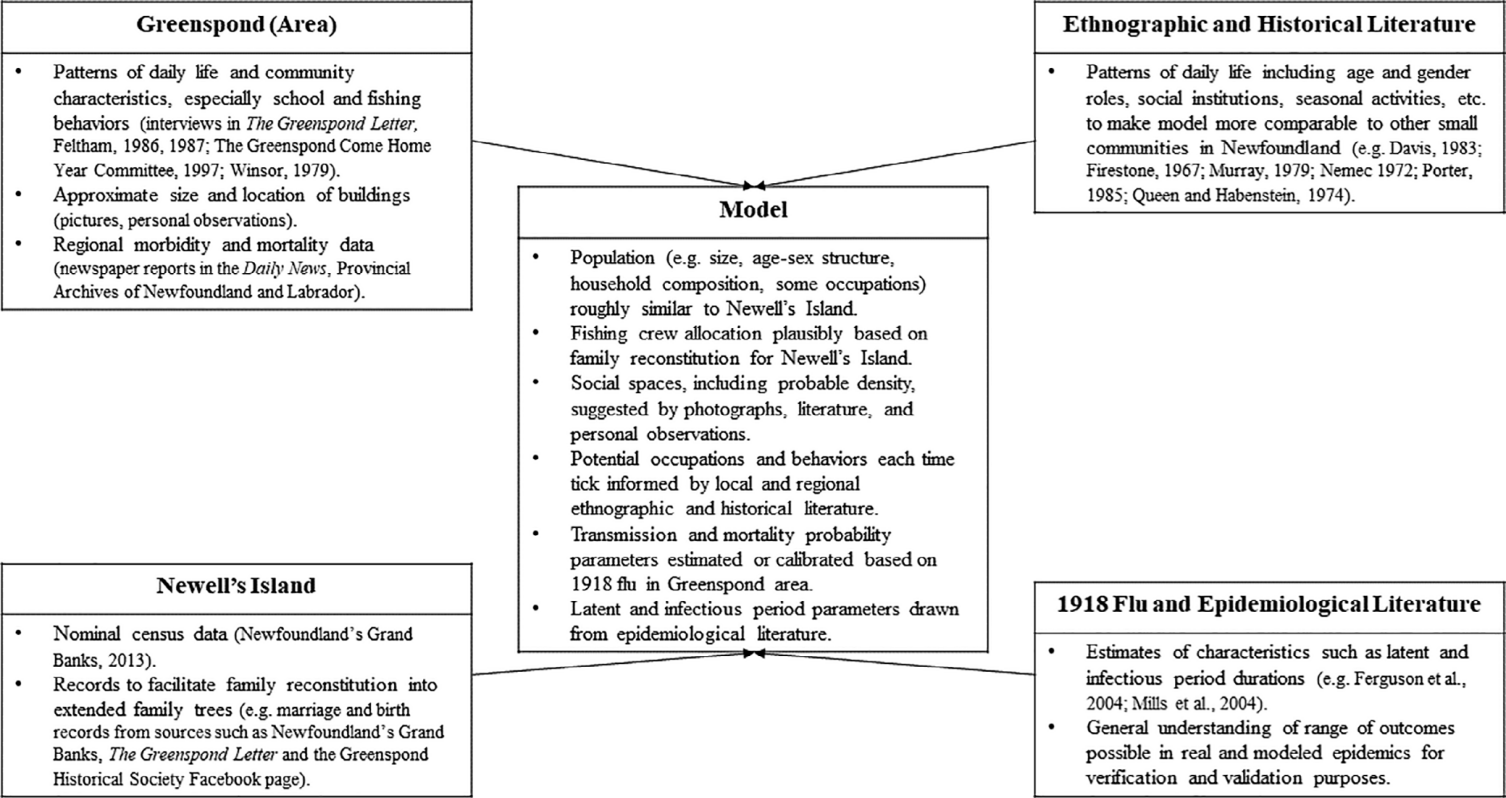
Types of sources and their incorporation into the model design

Compared to mathematical models commonly used in epidemiological research, agent‐based models can be more intuitively built and communicated in terms of realistic, if simplified, behaviors and social spaces. They also allow for the relatively easier inclusion of historical, archival, and ethnographic data. Our agent‐based model was constructed in NetLogo 5.0.3 (Wilensky, 1999). Figure 2 illustrates how a variety of sources (including historical, ethnographic, and archival material for the Greenspond region and Newfoundland in general; genealogical information for Newell's Island in particular; and literature on the 1918 flu and epidemiological theory) were combined to develop a model community that is grounded in realistic details but is not an exact replica of Newell's Island. Indeed, several features of the model, such as the presence of churches on the island itself, are not based in reality.

This approach was taken for several reasons. First, although there is a wealth of historical information for the Greenspond area, it is also unusual due to its size and economic and political importance. Modeling Newell's Island specifically allows us to use available and relevant historical material while also representing a more typical community. Second, the process of family reconstitution to connect extended families and allocate fishing crews would have been prohibitive for the entire Greenspond region. Most importantly, we do not intend to recreate what actually happened during the 1918 pandemic in the study community. Rather, detailed information is used to construct plausible yet generalizable scenarios to test hypotheses about disease spread in communities that share similar characteristics, such as small size, kinship organization around economic activities, and for these analyses, school structure and practices.

In the interest of space, we briefly describe the model here and provide an extensive description in the supporting information. The NetLogo model is also available upon request (see also Sattenspiel, 2019 for a similar model). The model population is based on the 1911 nominal census for Newell's Island, which included names, sex, relationship to the head of the household, marital status, birth month and year, age, and birthplace for 114 people in 23 dwellings. We used available data and reasonable assumptions to link together groups with the same surname into an extended kinship network. The identified relationships were used to assign characteristics to the agents in the model, such as membership in kin‐based fishing crews and church congregations.

**FIGURE 3:**
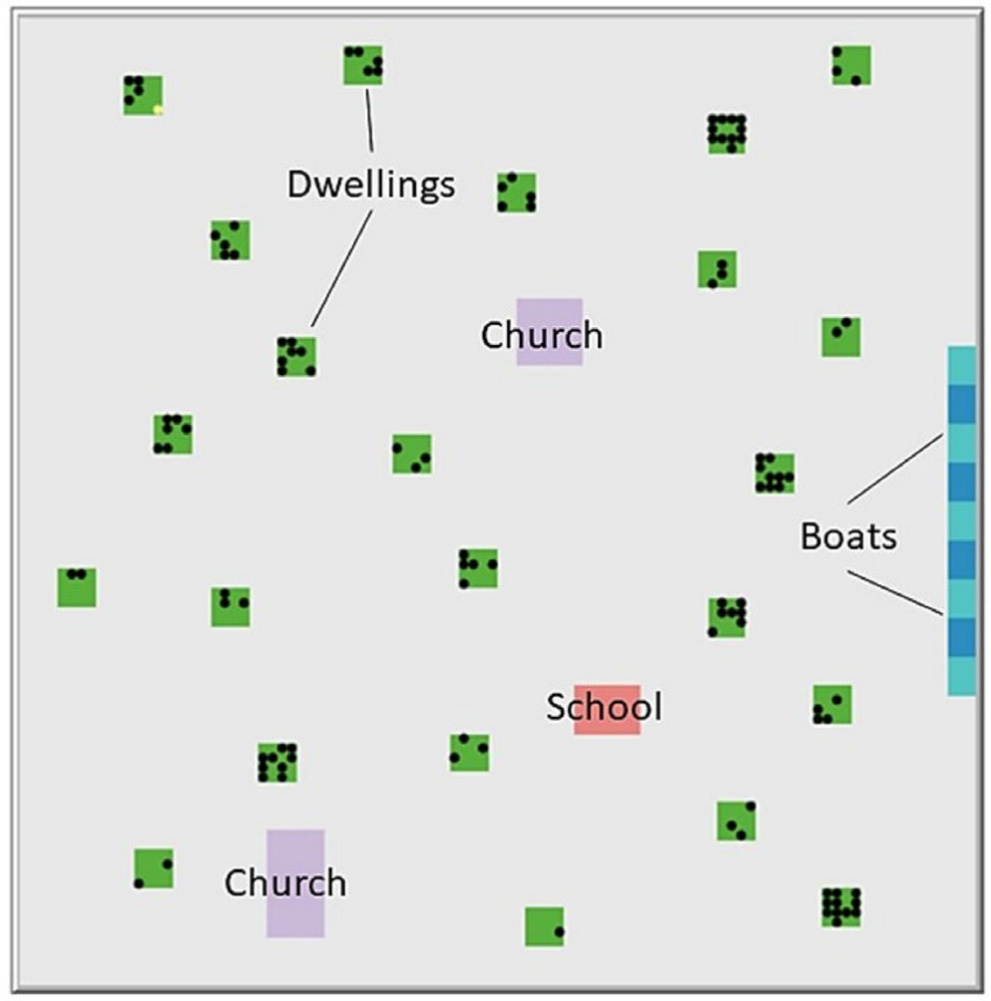
Visualization of the agent‐based model at initialization

The model space contains dwellings, a school, churches, and boats, which also represent shore crews (Figure 3). Agents begin a simulation in their dwelling at 6:00 a.m. on Monday. There are five basic processes, which agents complete in a random order each time step, representing 4 h each. First, each agent updates its disease status; for example, exposed agents switch to infectious when they have completed the latent period. Second, agents typically move within or to a new social space determined by the day and time period and the agent's occupation. Once they move, disease transmission may occur between neighboring susceptible‐infectious pairs. After these steps are completed, reassignment might occur to replace any newly deceased agents who were teachers, clergy, or primary caretakers for children. Finally, the model updates its output files and the plots and monitors on the interface.

Most agents have a primary activity each day, although some can choose among different options with some probability. Table 1 provides an overview of the activities of the different types of agents Monday through Saturday in the baseline model. On Sundays, all agents behave the same: everyone attends their assigned church during the first daytime step, before returning home for the second step. In the next step, each family may choose to visit another dwelling or remain at home.

**TABLE 1:** Primary activities of different agent types during model simulations

Agent type (*n*)	Monday–Friday activity	Saturday activity
Fishermen (41)	Fishing boat	Fishing boat
Fisherwomen (Adult women [>15] with no children or children ≥10 only) (17)	0.8 chance of working in shore crew, else visit other households, or stay home.	0.8 chance of working in shore crew, else visit other households, or stay home.
Adult women without preschool children but at least one child aged 5–10 (0)^a^	0.8 chance of working in shore crew, else visit other households, or stay home.	0.5 chance of visiting other households, else stay home. Children <10 may accompany them.
Adult women with at least one preschool child (13)	0.5 chance of visiting other households, else stay home. Preschool children accompany them.	0.5 chance of visiting other households, else stay home. Preschool children will, and school‐aged children <10 may, accompany them.
Teachers (2)^b^	School	0.5 chance of visiting other households, else stay home.
Traveling clergy (1)	Travel outside the community (cannot be infected).	Travel outside the community (cannot be infected).
Schoolchildren (21)	School	If ≥10 years, 0.8 chance of working on boat/shore crew. Otherwise, 0.8 chance of “playing” at school, else stay home. If <10 and do not accompany mother/caretaker, 0.8 chance of “playing” at school.
Preschool children (19)	Move with mother/caretaker	Move with mother/caretaker

^a^ No agents begin in this category, but a female agent can assume this occupation if she becomes the primary caretaker of school‐aged children whose previous caretaker has died.

^b^ One teacher is also pastor of one of the churches on Sundays.

Disease transmission can only occur between agents who form a susceptible‐infectious pair, are in the same social space or in adjacent boats, and are neighbors, that is, one agent is in the cell to the north, south, west, or east of the other. An infectious agent determines whether it has any susceptible neighbors, and if so, a random number uniformly distributed between 0 and 1 is compared to the transmission probability parameter to determine whether transmission occurs. An analogous process occurs when susceptible agents search for infectious neighbors. A newly infected agent enters the exposed disease category and proceeds through the infectious and to the recovered stage based on the duration of the respective parameters. In the model, only infectious agents may die, with the probability of dying equal across all time steps of the infectious period.

### 2.3 School attendance scenarios

In the baseline model, all children aged 5–15 attend school every weekday. Two alternative scenarios were modeled to consider different patterns of school attendance. In the Skip model, children aged 10–15 attend school each day with some probability, or else engage in fishing activities. In the Reassign model, a proportion of the children aged 10–15 are reassigned to fishing activities for the entire duration of the simulation; the proportion is larger for children >12 to reflect an increased likelihood of such behavior with age. The probabilities used to determine school attendance in both models are varied across a range of values from 0 to 100 percent.

A total of 500 simulations were run for each scenario, but analyses were only performed on runs producing epidemics, defined as >5% of the population ever infected. Disease parameters used in the simulations reflect 1918 influenza estimates and are constant for all agents throughout each simulation (Table 2). Analysis of variance (ANOVA) was used to compare outcomes, including the average proportion of the population ever infected (final size), the average proportion of infectious agents at the peak (peak size), the average day (i.e., number of time steps divided by 6) of the peak, and the average last day any agent was infectious.

**TABLE 2:** Model parameter values estimated to reflect the 1918 influenza pandemic

Parameter	Value	Notes
Latent period	1 day (6 ticks)	Informed by 1918 flu literature.^a^
Infectious period	3 days (18 ticks)	Informed by 1918 flu literature.^a^
Transmission probability per contact	0.045	Determined via preliminary sensitivity analyses to produce an attack rate of about 30%, the observed morbidity for the Greenspond region.
Mortality probability per tick of the infectious period	0.0013	Estimated from the observed mortality for the Greenspond region (about 7.3 deaths per 1000). See the supporting information for the calculations used to derive this value.

^a^ For example, Ferguson et al., 2004; Mills et al., 2004.

## 3 RESULTS

**FIGURE 4:**
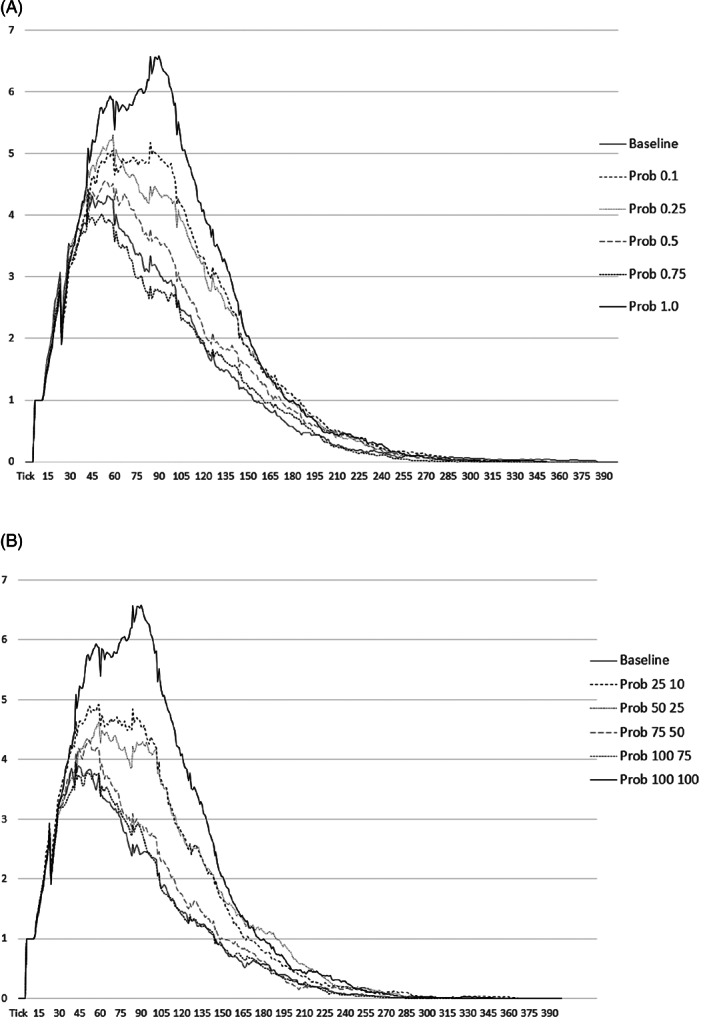
Average epidemic curves for each attendance probability in the (A) Skip and (B) Reassign scenarios

For both scenarios, comparisons of means across all probabilities of attendance demonstrate statistically significant differences (Tables 3 and 4). As Figure 4 and these tables illustrate, the general trends are that the average final size decreases, there are fewer cases at the peak, and the peak day occurs earlier as school attendance decreases. Generally, results are slightly more marked in the Reassign model, where more of the population consistently escapes infection than in the Skip model. Both scenarios have a larger effect on epidemic size than timing. For example, the final size drops by approximately half in the Reassign model, while the peak is ~3 days earlier (a decrease of about 21%), with maximum reassignment compared to the baseline.

**TABLE 3:** Epidemic outcomes in the Skip model, varying daily probability of children aged 10–15 fishing instead of attending school

Probability of fishing each day (%)	No. epidemics	Final size (% of population ever infected)	Peak size (% of total population)	Peak day	Last day
0 (Baseline)	234	0.35	0.11	13.9	25.5
10	243	0.30	0.09	13.4	24.9
25	232	0.29	0.09	12.5	24.3
50	246	0.25	0.08	12.4	22.7
75	254	0.21	0.07	11.5	21.2
100	231	0.22	0.07	11.3	21.5
ANOVA (F_5,1434_)		21.21*	26.92*	5.71*	7.99*

^*^ *p* < .05.

**TABLE 4:** Epidemic outcomes in the Reassign model, varying probability of reassigning children to fishing occupations for the entire simulation

Probability of reassignment (%) 13–15 years | 10–12 years	No. epidemics	Final size (% of population ever infected)	Peak size (% of total population)	Peak day	Last day
0 | 0 (Baseline)	234	0.35	0.11	13.9	25.5
25 | 10	235	0.28	0.09	12.8	23.2
50 | 25	228	0.27	0.08	13.2	24.1
75 | 50	225	0.21	0.07	11.6	20.8
100 | 75	246	0.19	0.06	10.8	20.1
100 | 100	247	0.18	0.06	11.0	20.2
ANOVA (F_5,1409_)		34.84*	40.78*	9.40*	13.5*

^*^ *p* < .05.

Analyses of the Reassign model suggest that all school‐aged agents are more likely to escape infection as the probability of reassignment increases. However, those students who remain in school have a slightly higher likelihood of becoming infected than students who are reassigned, at least with moderate probabilities of reassignment overall (Table 5). The reduction in final size is not simply equivalent to the number of children removed from school. Additional agents escape the epidemic when older children engage in adult occupations rather than attending school. These differences are relatively minor and show no trend with increasing probabilities, but they nonetheless indicate marginal benefits at the community level that might occur with atypical school attendance (Table 6).

**TABLE 5:** Average percentage of epidemics escaped by school‐aged agents when reassigned to adult occupations vs. when attending school

Probability of reassignment (%) 13–15 years | 10–12 years	Average percentage of epidemics escaped by a reassigned student	Average percentage of epidemics escaped by a student who attended school
0 | 0 (Baseline)	‐	0.49
25 | 10	0.68	0.60
50 | 25	0.69	0.61
75 | 50	0.76	0.74
100 | 75	0.78	0.78
100 | 100	0.78	‐
ANOVA	F_4,70_ = 4.67*	F_4,62_ = 29.25*

^*^ *p* < .05.

**TABLE 6:** Excess impact on final size beyond the number of reassigned students

Probability of reassignment (%) 13–15 years | 10–12 years	Average final size	Difference from baseline	Average number of students reassigned	Average number of additional agents who escaped infection relative to baseline
0 | 0 (Baseline)	39.5	‐	‐	‐
25 | 10	31.6	7.9	2.8	5.1
50 | 25	31.0	8.6	5.6	3.0
75 | 50	23.6	15.9	9.8	6.1
100 | 75	21.2	18.3	13.4	4.9
100 | 100	21.1	18.4	15.0	3.4

## 4 DISCUSSION

The removal of children from school has significant effects on epidemic size and timing in both of the modeled scenarios. Consistent with other work (e.g., Fumanelli et al., 2016; Jackson et al., 2013), these results demonstrate the importance of school attendance in disease spread within the community. Interruptions to routine school interactions, therefore, have the potential to affect epidemic outcomes for children, their families, and members of the larger community. In these analyses, there are benefits for school‐aged children, regardless of whether they were among the reassigned individuals. However, consistent with mixed results in previous literature (e.g., Cauchemez et al., 2011; France et al., 2010), it is unclear how much changes in school practices alone protect non‐school‐aged individuals in the population.

Each simulated week has 42 ticks, with Saturday and Sunday corresponding to ticks 31–42, 73–84, and 115–126 in the first 3 weeks, and so on. Effects of weekend behavior similar to those discussed by Towers and Chowell (2012) are not immediately apparent in the simulated epidemic curves (see Figure 4), perhaps because there is still considerable contact between different households due to fishing and children playing together on Saturday, church attendance on Sunday, and visiting both days. Due to the stochastic nature of the model and the small population size, the curves are quite jagged, making it difficult to observe potential weekend variation. Nonetheless, the apparent lack of weekend effects in the current study suggests that the dominant school attendance behaviors during the week likely have a larger impact on epidemic spread than the regular weekend behavior.

### 4.1 Limitations

In addition to potential limitations discussed more below, these analyses do not consider children staying home sick. The decision‐making process guiding such behavior of either students or their adult caretakers includes both barriers and incentives and is likely to be different from the processes influencing non‐health‐related school attendance decisions. However, most disease models only consider pathogen transmission and not symptom presentation, and considering potentially high rates of asymptomatic or minor cases, it is reasonable to assume regular behavior of many agents even when infected. Future work will assess the impact of health‐related behavior change in this model community.

We also do not consider gender‐based differences in school attendance. In the model, there is a balanced sex ratio among the older children (seven boys, eight girls). All children have the same probability of attending school, and if they do not attend, all children are reassigned to fishing occupations. While this assumption is reasonable for irregular absences, such as during fishing seasons, girls who permanently left school at early ages might have done so for service occupations or domestic labor including care of younger siblings or marriage. Exact rates of leaving school are unknown for this community, so reassignment to fishing activities is treated as a proxy for all possible alternatives. Like other fisherwomen in the model, permanently reassigned girls have some probability of staying home or visiting other households, which could reflect other employment or domestic roles. In the model, replacement caretakers for children orphaned by the epidemic are only drawn from adults.

### 4.2 Implications for historical and present‐day pandemics

Our findings may help explain observed patterns in Newfoundland during the 1918 pandemic. Out of 17 districts, only five had higher mortality rates than that of the capital, St. John's (Sattenspiel, 2011). Although many factors likely contributed to this variation, less frequent school attendance in more rural locations might be one. There is limited evidence for school closures during the pandemic, but correspondence with the Colonial Secretary's office verified that newspaper notices regarding closing places of public gathering were only applicable to St. John's, while officials in other communities were responsible for similar orders (Provincial Archives of Newfoundland and Labrador, 1918). Even if formal public health measures were not taken by local authorities, our results suggest that school attendance patterns alone might have led to relatively smaller epidemics outside of the major population centers.

Further, these findings have relevance for COVID‐19 and future pandemics. This study demonstrates that, in communities where daily or seasonal factors result in absences or where children assume other productive roles, epidemics are likely to be smaller and shorter than would be otherwise expected. The shorter epidemics are of particular note, because one goal of school closures is to delay peaks and buy time for the implementation of other public health responses. Although the absolute difference in timing was small compared to the differences in size, these findings reinforce the need to consider varied types of educational practices to determine whether school closures or other interventions are likely to produce the intended effect. The reassignment scenario is comparable to a situation where some but not all parents choose virtual learning or homeschooling for an extended period, while the daily attendance scenario is similar to strategies where students attend alternating or partial days. Our findings suggest that this latter approach would have a slightly smaller effect on controlling an epidemic.

All agents in the model are equally susceptible to infection, and all become infectious once exposed. In real epidemics, underlying health and other factors play a role in determining individual outcomes, and these often follow age‐specific trends. Children aged 5–14 tend to suffer lower mortality from infectious diseases and in general than other age groups (Mamelund et al., 2017), while Lau et al. (2015) estimated that children were approximately twice as infectious and three times as susceptible to influenza as adults. Early analyses of COVID‐19 suggest that children tend to have milder or asymptomatic cases, but unlike with influenza, they do not appear to play significant roles in transmission, although this is still uncertain (e.g., Dong et al., 2020; Heavey et al., 2020; Rajmil, 2020). Between March 1 and September 19, 2020, there were more laboratory‐confirmed cases among children aged 12–17 (63%) than those aged 5–11 (37%) (Leeb et al., 2020). The likelihood of leaving school for economic reasons increases with age, so if transmission more readily occurs among adolescents, the students who do not attend school would contribute to community transmission more than they do in our current results.

Further, populations in low‐ and middle‐income countries have higher rates of underlying health conditions and other infectious diseases, which may interact with pandemic diseases like COVID‐19, as well as less access to clean water and other resources. These populations are also disproportionately disadvantaged by school closures compared to high‐income countries (Zar et al., 2020). For schools like the one in our model, that is, ones that are small, have irregular attendance, and have relatively few adult employees who might be at higher risk, it might be more beneficial to keep schools open, especially if the transmission is shown to be relatively low for the relevant age groups.

Vaccination was not possible for the 1918 influenza pandemic nor immediately available for COVID‐19, and this is likely to be the case for future pandemics as well. If or when vaccines become available, however, calculations regarding required coverage, as well as the success of school‐based immunization programs, may be hindered by inaccurate assumptions about school attendance. Studies should explicitly consider contacts among homeschooled children, minors who do not attend any form of schooling, and others in the community. The most common reasons for school absence also need to be considered in governmental relief plans during pandemics. For example, income supplements should account for the perhaps unpaid or unofficial labor of children that contributes to family income.

These analyses demonstrate the need for data collection and modeling studies to sufficiently take into account absenteeism and dropout rates among students, particularly how they might vary in predictable ways for different socioeconomic groups, in areas with fewer services and resources, or seasonally. In evaluating responses to pandemics and preparing for future ones, it is important to consider the wide range of variation in educational strategies that might produce atypical attendance or student demographics, including homeschooling, year‐round curricula, and charter schools, as well as public health strategies that do not involve full closure. Future work must continue to consider the social, cultural, economic, and ecological factors that determine attendance and by extension the activities of school‐aged children and their interactions with each other and other members of their households and communities.

## AUTHOR CONTRIBUTIONS

**Jessica Dimka:** Conceptualization; formal analysis; methodology; software; writing‐original draft; writing‐review & editing. **Lisa Sattenspiel:** Conceptualization; methodology; software; writing‐original draft; writing‐review & editing.

## CONFLICT OF INTEREST

The authors have no conflicts of interest to declare.

## Supporting information


**Appendix S1**: Supporting Information

## Data Availability

The data used in these analyses are results produced by stochastic model simulations. The model code and files are available upon request from the authors (see also references in the manuscript and supporting information).
