# Supplementary material for: “We didn't get much schooling because we were fishing all the time”: Potential impacts of irregular school attendance on the spread of epidemics
Source: Am J Hum Biol. 2021 Feb 17;34(1):e23578. doi: 10.1002/ajhb.23578 (PMC7995059; doi:10.1002/ajhb.23578)
Supplement: Supplementary file 1 — Appendix S1: Supporting Information [file AJHB-34-0-s001.docx]

**Supporting Information – Model Description and Protocol^[[1]](#footnote-1)^**

**Overview**

Purpose: The aim of the model is to investigate how social institutions and interactions influence disease spread within a small, kin-based community – specifically, a fishing village in Newfoundland – around the turn of the 20^th^ century. The 1918 influenza pandemic is used for realistic context, but results of the model could be generalized or compared to other similar areas, time periods or diseases with minor modifications. Research questions include:

1. Which particular behaviors (e.g. fishing, school, church) contribute to larger or longer epidemics?
2. How do outcomes differ among subgroups of the population, given traditional gender and age roles?
3. Do public health interventions or individual health-related behavior changes influence the likelihood, size or duration of epidemics?

NetLogo files and code are available from the authors upon request.^[[2]](#footnote-2)^

Entities, state variables and scales: The model environment depicts a simplified village, including 23 dwellings, a school, two churches, and nine boats that also serve as the shore crew workspace (Figure S1). Different patch colors indicate the various social spaces, and patch variables include the type of space and an identification number for the specific location. Because exact locations of dwellings and other locations in the study community are not available from archival sources and because research goals emphasize transmission within important social spaces rather than transmission due to encounters while traveling between such spaces, the positions of the buildings do not reflect geographical reality. The sizes of the respective building types are chosen to approximate reasonable densities based on estimated sizes of such buildings in the community and their typical occupancies.

**
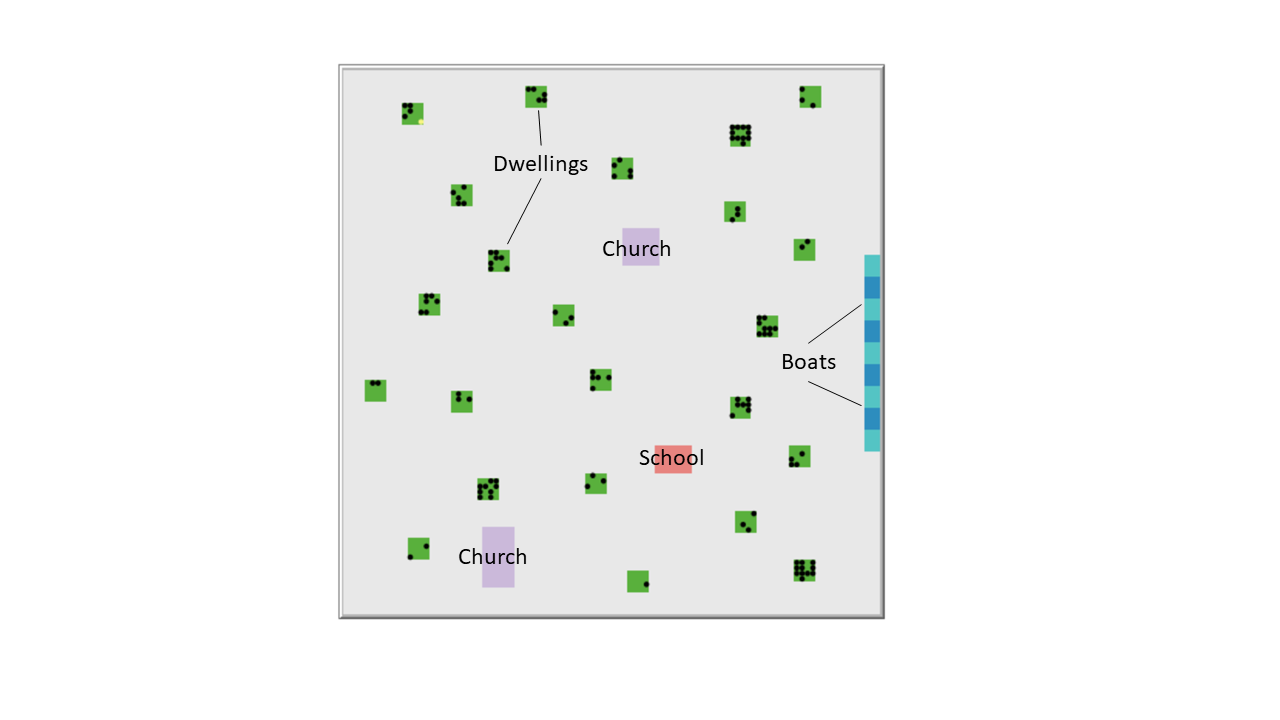
**

**Figure S1.** Visualization of social spaces in the model environment.

The model population is based on the individuals listed in the 1911 nominal census for Newell’s Island, Newfoundland and Labrador, which included full names, sex, relationship of the individual to the head of the household, marital status, birth month and year, age, and birthplace for 114 people distributed into 23 dwellings. Additional information from sources such as parish records and published interviews further support the genealogical details provided by the census. Whenever possible, multiple sources were used to link individuals and households together, especially when slight variations in dates or spelling of names occurred.

The first phase of family reconstitution focused on family relationships that were considered reliable from the preponderance of available data. In the second phase, reasonable assumptions were made to link together groups with the same surname into larger family trees. Typically, these assumptions either were based on references in various secondary sources to individuals with no corresponding records or involved choosing among a small number of possible individuals when records were insufficient to determine which one was the actual relative referenced in different sources. Decisions were reinforced by ages or dates of relevant life events; for example, potential parent-child relationships were assumed to be rational if the age difference between the two individuals fell within a biologically possible and culturally reasonable range for reproduction. These strategies resulted in a few large family trees that connected the majority of households in the census into an extended kinship network. The identified relationships were then used to assign characteristics to the agents in the model population.

Table S1 summarizes the major agent attributes. In the model, agents are represented by circles that change color depending on disease status.

Each time step of the model represents 4 hours, and each simulation begins on Monday at 6 AM, then proceeds to run for 400 ticks (approximately 67 days) to allow sufficient time for most epidemics to end. Table S2 summarizes global variables including the parameters that govern disease transmission processes.

**Table S1.** Agent Attributes^1^

| Variable | Notes |
| --- | --- |
| Agent ID |  |
| Residence | All agents are set to 1, which corresponds to Newell’s Island. The variable is intended for potential future expansions that consider multiple communities. |
| Disease status | Susceptible (0), Exposed (1), Infectious (2), Recovered (3), Dead (4) |
| Dwelling | ID number |
| Household | ID number. Multiple households per dwelling are possible. |
| Extended family | The ID of an agent’s extended family, currently used in adoption methods only. The values correspond to surnames listed in the 1911 census. |
| Sex | Male (0), Female (1) |
| Age | Calculated from birth information in census and archival records. Age-related differences in susceptibility or severity of disease outcomes are not considered. |
| Church | The members of each household were assigned to one of two churches in the model. Records indicate that Newell’s Island residents adhered to three major denominations (Church of England, United/Methodist and Salvation Army), but only churches for the first two denominations are considered in the model, partly because of the relatively small congregation size identified for the Salvation Army. Individuals whose records indicated probable membership in the Salvation Army were assigned to the other two churches along family lines if possible, as well as to maintain distributions proportional to those indicated for Newell’s Island in the 1921 aggregate census.  Church assignments were generally informed by archival records, such as baptism, marriage and burial records. In these records, religion of the individuals was explicitly listed or can be inferred from the name or affiliation of the person performing the rites. When such information was unavailable, individuals were generally assigned to the same churches as close family members in different dwellings. |
| Health history | Corresponds to an agent's relative health status, designed to take into account different possible influences that may impact an agent's outcome when faced with a potential disease-transmitting contact. This variable can range from -1 to 1, with -1 corresponding to a maximum negative impact (i.e., 100% reduction), 0 corresponding to no impact on health, and 1 corresponding to a maximum positive impact. All agents are currently set to 0 because this variable is designed for future model versions. |
| Occupation | Agent behavior categories that relate to normal daily activities, often corresponding to age and sex. The variable is a 3-digit code, with the first digit corresponding to an occupation type (e.g. 1 = fisherman) and the other digits corresponding to the boat-ID. See below for more details on how this variable is used. |
| Boat ID | Individuals in the model population were assigned parsimoniously to fishing crews according to crew formation strategies described in the ethnographic literature. Crewmates typically included fathers, sons, brothers, and cousins who were grouped together until target crew sizes of approximately 4-8 men were achieved; this target is supported by photographs, published recollections, and personal communication with other researchers. The model population is thus distributed into nine crews actually ranging in size from 4 to 6 men. All agents within a household are assigned the same boat ID, regardless of the individual agent’s specific occupation. |

1. Additional procedural variables are “owned” by agents in the model in order to facilitate specific processes or methods but are not included in this table. Examples include the coordinates of their intended destinations during movement methods, disease-related variables such as how many days remain before an affected agent become infectious or recovers, and variables for finding replacement or adoptive caretakers upon the death of a parent or guardian.

**Table S2.** Global Variables^1^

| Parameter | Default | Notes/Source |
| --- | --- | --- |
| Transmission probability per contact | 0.045 | Estimated/calibrated to achieve a target 30% attack rate, based on empirical observations of flu experience in the broader Greenspond region (at least 400 cases out of approximately 1500 people). |
| Mortality probability per tick of the infectious period | 0.0013 | Estimated from mortality data for the broader Greenspond region, and derived by setting the desired total survival probability equal to [(1- the probability of death per time step) raised to a power corresponding to the number of time steps that an agent is at risk of dying]. This quantity gives the overall probability of not dying throughout the infectious period, so it is subtracted from 1 and set equal to the desired case fatality rate. Solving the equation gives the probability of dying per time step. Mortality for Greenspond during the 1918 flu was about 7.3 deaths per 1000, which converts to a case fatality rate of about 24 deaths per 1000 using a 30% attack rate. Using an infectious period of 18 ticks or 3 days gives the equation:  1 – (1 – probability of death) ^ 18 = 0.024  The solution to this equation gives the default mortality probability per tick. |
| Length of latent period | 1 day (6 ticks) | Literature on 1918 flu^2^ |
| Length of infectious period | 3 Days (18 ticks) | Literature on 1918 flu^2^ |
| Church density | 1.0 | Proportion of the available space within a church allowed to be filled during a service. Both churches are assumed to use the same density. |
| First case ID | Integer |  |
| First case occupation | Integer |  |

1. Additional procedural variables are included in the model in order to facilitate specific processes or data collection but are not included in this table. Examples include the population size and the number of agents in each disease status, the number of ticks a simulation runs, and a tracker variable to determine the six-hour time period corresponding to the current tick.
2. e.g. Ferguson, Fraser, Donnelly, Ghani, & Anderson, 2004; Mills, Robins, & Lipsitch, 2004

Process overview and scheduling: Five major processes can occur each time tick, described in more detail below. First, each agent updates its disease status, e.g. an exposed agent switches to infectious when it has completed the latent period. Second, agents typically move within or to a new social space (e.g. home, boat, school) corresponding to the day and time period represented by the time tick and determined by the agent’s occupation. Once they move, neighboring susceptible-infectious pairs of agents might transmit the disease. After these actions are completed, if any agents died that time step, reassignment might occur to identify new caretakers for orphaned children or to fill vital roles in the community. Finally, the model updates its output files and the plots and monitors on the interface.

**Design Concepts**

Basic Principles: Theoretical concepts underlying this model include, in a broad sense, approaches such as social determinants of health. Social interactions and contact patterns that lead to disease transmission and epidemic outcomes are strongly influenced by economic and cultural factors, such as traditional gender roles. Local cultural contexts can help explain why outcomes observed at regional and smaller scales can be highly variable within the context of a larger epidemic. This model specifically considers how the historical development of outports (i.e. small communities along the coast) in Newfoundland centered around fishing and related industries led to behaviors and ways of life that might have influenced disease spread. Despite this focus, findings from the model can also be extended to communities with similar size or structure (i.e. typically nuclear households and kin-based organization of subsistence employment).

Emergence: Epidemics of flu will emerge in the community with some probability. The occurrence, size and timing of these epidemics are influenced by a variety of individual, social, epidemiological and random factors.

Adaptation: In the default model, there is limited health-related behavior change during the epidemics. Some individuals might take on additional roles or activities if another one dies, depending on the responsibilities of the dead agent. No behavior change occurs solely as a result of illness, however.

Sensing: Agents are able to access the values of their own disease-status variables, as well as the values of those variables for relevant neighbors, in order to determine whether disease transmission occurs. Note that the disease transmission model considers infectiousness of agents, not whether they are symptomatic.

Interaction: Heterogeneous behaviors or characteristics will bring some agents into close and/or frequent proximity with each other. For example, students spend a large portion of each day together. This direct interaction will influence disease transmission.

Stochasticity: The first case is selected randomly, although users can define other criteria. Agents proceed through each time step in a random order. Disease transmission and mortality are determined by comparing random numbers drawn from a uniform distribution ranging from 0 to 1 to user-designated constant probabilities for the disease transmission and mortality parameters. The default model assigns each parameter a constant value at initialization. Destinations are determined by the schedule, but sometimes (such as when visiting), agents can choose from among a set of potential activities or destinations. Further, each agent will randomly select an unoccupied cell when it moves to a new location. Therefore, which agents are neighbors and thus are capable of transmitting disease to each other is stochastic.

Observation: Data output files always include basic information such as run number and initial and/or constant parameter values. The primary output consists of disease-related outcomes, including the number of affected agents per tick as well as the number of total cases and deaths per simulation. Another output file also records data on individual attributes and infections (e.g. when a specific agent was infected, where and by whom), so that impacts on different demographic subgroups can be evaluated. Visualization on the interface includes the changing colors of agents corresponding to disease status and the epidemic curve plot.

**Details**

Initialization: The default setup process begins with establishing the visualization and details of the community (model world) and the agents. Details are read in via a file that contains X and Y coordinates for the lower left corner, width and length dimensions, type and ID numbers for patches that designate different social spaces. Patch colors are chosen to differentiate the spaces visually. While the model population and appearance are generally informed by records and photographs for the Greenspond region and Newell’s Island specifically, the model is not meant to fully recreate any particular village, either spatially or demographically.

An agent attribute file is also read in. A new agent is created, with corresponding attributes assigned (see Table S1), until the model population is complete. Visualization details (size, shape, color) are set, and agents are moved to their dwellings, where they begin each simulation.

Global variables, including population trackers and the timekeeper, are initialized. The disease-related parameters are all set by sliders on the interface and so do not need to be initialized within the code itself.

One of the agents is then selected as the first case, and its disease-related variables and color are updated accordingly.

Finally, files to record output are created. Two files are updated at the end of each simulation. The “Cases” file collects information on each agent, such as whether it was infected, by whom and when. The “Final” file collects data on the total number of cases and deaths. The “Daily” file is updated each tick of a simulation with information on the current number of agents in each disease category.

The last action in the setup method is to start the timer.

Input Data: Not applicable.

Submodels:

*Update disease status:* Each time tick, each agent in the exposed category will check whether it has reached the end of the latent period and so should become infectious. If not, it will reduce its remaining time by one tick.

Infectious agents first have a chance of dying (and only infectious agents can die). A random number uniformly distributed between 0 and 1 is compared to the mortality probability parameter to determine whether death occurs. Agents who die change their disease-status accordingly and also set relevant variables to be recorded in the output file. They are effectively removed from the model population, but for visualization purposes, the agent object remains in the world and can be either hidden or visible in a ghost shape.

A surviving infectious agent then decides whether it should recover, based on the length of the infectious period, or reduce its remaining time as infectious by one tick.

Agents move from susceptible to exposed status as part of the transmission method, so no updates are needed for those agents in this submodel. Recovered agents continue regular activity through the rest of the simulation, but never become susceptible again.

*Movement methods:* The general movement process is as follows: based on the time period and agent category, an agent selects its desired destination. A random cell is selected from the subset of unoccupied patches corresponding to that social space, and the agent sets its location as the center of that patch. Only one agent is able to occupy a particular patch. Although such an occurrence is rare given the size of the buildings, if there are no available patches in the desired destination, the agent instead remains where it is. An exception is if the desired destination is the agent’s home from another location (i.e. if the agent is only supposed to move within its own home, it will stay where it is). To avoid unrealistic scenarios (such as a child staying overnight in the school), an agent who cannot find a place at home will pick a random house to visit until it moves again. When agents are visiting other households during the day, restrictions are placed so that the destination dwelling is selected from among the subset of houses that have enough space for the entire visiting party.

The detailed schedule of different types of agents is informed by ethnographic, historical and archival sources. While the exact durations or likelihoods of particular activities might not reflect reality, the general pattern of behaviors is designed to be plausible based on, for example, the agent’s sex and age (see Tables S3-S5 below).

*Transmission methods:* Following movement each time tick, any agent who is susceptible checks to see if it has any infectious neighbors in the cells to its north, south, east and west. If so and if a random number uniformly distributed between 0 and 1 is less than the transmission probability parameter, the susceptible agent changes its disease status to exposed and updates related variables. An analogous process takes place for infectious agents who look for susceptible neighbors. There are controls in the code to prevent attempts at transmission to agents who were newly infected earlier in the same time step. Agents must be in the same social space or in adjacent boats for transmission to be possible.

*Reassign-Occupation methods:* If an agent dies during a time step, another agent might assume its duties, such as caring for dependent children. If a dying agent is older than 15, replacement will dependent on its occupation. Fisherwomen (occupation type [occ-type] 0 or 2) or mothers (occ-type 7) with surviving children will seek a replacement caretaker. Fishermen (occ-type 1) might be assigned to caretaker roles if they are the only surviving adult in the household; if so, dying agents in this role will seek a replacement caretaker for any surviving children. Agents first look for potential replacements among members of their household, then dwelling, then extended family, and then any suitable adults in the community. Within these categories, the preference is first an agent of occ-type 0 or 7, then 2, then 1, then 3 (teachers); teachers can become caretakers for school-aged children only. If no caretaker replacement is identified, children are assumed to be adopted “out” of the community, although this would be a highly rare occurrence (i.e. virtually all the adult agents would have to die before the end of the simulation). If the new caretaker is a male who becomes responsible for preschool-aged children, he becomes a stay-at-home-dad. He retains his fisherman occupation but does not move to the boats as long as he continues to be responsible for children under age 5. Fishermen-caretakers of older (school-aged) children continue to engage in their regular activities, however.

If the dying agent is younger than 15 and its primary caretaker is an adult male, the child will determine if it has any surviving siblings. If not, the adult male will lose its caretaker status, or if there are only school-aged siblings who are surviving while the dying agent was younger than 5, the adult male will switch from a stay-at-home-dad to a fisherman-caretaker. Female caretakers retain their current occupation status for the remainder of the simulation, even if all their dependent children have died.

Similarly, dying teachers are replaced by suitable living agents. As the male teacher is also a pastor in this model community, it chooses potential replacements from among fishermen who are older than 30 years old, from the same church congregation, and who are not currently stay-at-home-dads. The chosen fisherman's boat must have at least 3 fishermen assigned to it prior to reassigning the replacement, so the crew is left with at least 2 men. Because teacher-pastors can also be caretakers for school-aged children, they must find a new caretaker for any children under their care. The new caretaker and new teacher-pastor do not have to be and will likely not be the same agent. Female teachers who die choose a replacement from among fisherwomen (occ-type 2).

Finally, the other pastor in the community is considered traveling clergy. Pastors were known to travel circuits in the region to visit and care for more dispersed congregants. To account for this activity, and so the number of teachers is not overrepresented, the pastor of the second church, a 27-year-old male at initialization, leaves the model community during daytime hours on Monday-Saturday. Therefore, he can only come into contact with other community members on Sundays. Currently, the model does not allow for him to have a chance of infection during his travels, i.e. he can only be infected through contact with others in the study community or if he is selected as the first case. Further extensions of this model may test the effects of allowing him to get infected during his travels or of systematically choosing him as the first case who brings the disease into the community. If this agent dies, it chooses potential replacements from among fishermen who are older than 25 years old, from the same church congregation, and who are not currently stay-at-home-dads. As with the other pastor, the chosen fisherman’s boat must have at least 3 fishermen assigned to it prior to reassignment. This occupation allows slightly younger replacements, because it is assumed that a person in this general role may be a missionary or early in his career. Further, replacements are restricted to agents who do not have childcare duties, and similarly, the clergyman in this role is prevented from adopting orphaned children.

*Data collection and visualization methods:* At the end of each step, the tallies for population size and numbers of individuals in each disease status are updated. The relevant information is recorded to a new line in the “Daily” output file, and the plot on the interface depicting the epidemic curve is also updated. At the end of each simulation, the “Cases” file is updated, with each agent recording its relevant information in order by ID number, and the “Final” file is updated as well.

Verification and Validation: The model was verified and validated through several methods: 1) Model code was regularly and thoroughly tested during development to make sure it was correct and behaving as expected. 2) Output was compared to theoretical predictions and empirical observations of epidemics to make sure the model produces patterns consistent with expectations. For example, characteristic epidemic curves should be observed, and it should be possible for an epidemic to fail to take off, even under optimal conditions for disease spread. 3) Sensitivity analyses were conducted to determine the range of potential outcomes with different parameter values. 4) Repetition analyses were performed to determine the target number of simulations needed for a given parameter set to minimize “noise” and obtain a reliable average output. These analyses suggested that at least 500 simulations should be run to address research questions.

**Table S3**. Schedule indicating activity or destination corresponding to different time periods for different kinds of agents (Monday-Friday). Occ-types are in brackets.^1^

| **Time** | **Adult women (>15) without preschool children but at least one child aged 5-10 [0]** | **Fishermen [1]^2^** | **Fisherwomen (Adult women with no children or children ≥10 only) [2]** | **Teacher [3]** | **Traveling Clergy [4]** | **Adult women with at least one preschool child [7]** | **Schoolchildren [8]** | **Preschool children [9]** |
| --- | --- | --- | --- | --- | --- | --- | --- | --- |
| **6 - 10 AM** | Home | Boat (Home) | Home | School | Out of the community | Home | School | Home |
| **10 AM - 2 PM** | Unless hosting visitors, 0.8 chance boat, else 0.2 chance visit others, else home | Boat (Home) | Unless hosting visitors, 0.8 chance boat, else 0.2 chance visit others, else home | School | Out of the community | Unless hosting visitors, 0.5 chance visit others, else home | School | Move with mother/ caretaker |
| **2 - 6 PM** | If at boat, stay there. If visiting, return home. | Boat (Home) | If at boat, stay there. If visiting, return home. | School | Out of the community | Home | School | Home |
| **6 - 10 PM** | All agents move home | | | | | | | |
| **10 PM – 2 AM** | All agents asleep - no movement | | | | | | | |
| **2 – 6 AM** | All agents asleep - no movement | | | | | | | |

^1^ As noted above, this model is based on Sattenspiel (2019), which includes several other types of occupations not present in the Newell’s Island model. However, the occ-type numbering system was mostly retained, hence the gap between types 4 and 7. ^2^ If the agent is serving as a stay-at-home dad for preschool children, it does the activity in parentheses.

**Table S4**. Schedule indicating activity or destination corresponding to different time periods for different kinds of agents (Saturday)

| **Time** | **Adult women (>15) without preschool children but at least one child aged 5-10** | **Fishermen^1^** | **Fisherwomen (Adult women with no children or children ≥10 only)** | **Teacher** | **Traveling Clergy** | **Adult women with at least one preschool child** | **Schoolchildren** | **Preschool children** |
| --- | --- | --- | --- | --- | --- | --- | --- | --- |
| **6 - 10 AM** | Home | Boat (Home) | Home | Home | Out of the community | Home | If ≥10, 0.8 chance of boat, else home | Home |
| **10 AM - 2 PM** | Unless hosting visitors, 0.5 chance of visiting others, else home. Accompanied by any schoolchildren <10 who have not left independently earlier in the same time step. | Boat (Home) | Unless hosting visitors, 0.8 chance boat, else 0.2 chance visit others, else home. | Unless hosting others, 0.5 chance visit others, else home. | Out of the community | Unless hosting others, 0.5 chance visit others, else home. Accompanied by all preschool children and any schoolchildren <10 who have not left independently earlier in the same time step. | If <10 and not already accompanying mother/caretaker, 0.8 chance “play” at school.  If ≥10 and already at boat, stay there. Otherwise, 0.8 chance “play” at school, else home. | Move with mother/ caretaker |
| **2 - 6 PM** | Home | Boat (Home) | If at boat, stay there, else home. | Home | Out of the community | Home | If <10, home. Else, if older and already at boat, stay there, else home. | Home |
| **6 - 10 PM** | All agents move home | | | | | | | |
| **10 PM – 2 AM** | All agents asleep - no movement | | | | | | | |
| **2 – 6 AM** | All agents asleep - no movement | | | | | | | |

^1^ If the agent is serving as a stay-at-home dad for preschool children, it does the activity in parentheses.

**Table S5**. Schedule indicating activity or destination corresponding to different time periods for different kinds of agents (Sunday)

| **Time** | **Adult women (>15) without preschool children but at least one child aged 5-10** | **Fishermen** | **Fisherwomen (Adult women with no children or children ≥10 only)** | **Teacher** | **Traveling Clergy** | **Adult women with at least one preschool child** | **Schoolchildren** | **Preschool children** |
| --- | --- | --- | --- | --- | --- | --- | --- | --- |
| **6 - 10 AM** | All agents move to assigned church. There is no attempt to keep members of the same household “seated together”. The model assumes that the church is large enough to hold all assigned agents at the same time, that there is only service per church, and that all agents attend church. | | | | | | | |
| **10 AM - 2 PM** | All agents return home | | | | | | | |
| **2 - 6 PM** | The first adult in a household to go through the time step determines the activity and directs all other agents in the household to accompany them. Unless hosting visitors, 0.5 chance visit others, else home. | | | | | | | |
| **6 - 10 PM** | All agents move home | | | | | | | |
| **10 PM – 2 AM** | All agents asleep - no movement | | | | | | | |
| **2 – 6 AM** | All agents asleep - no movement | | | | | | | |

1. Broadly follows ODD protocol (e.g. Railsback, S. F. & Grimm, V. (2012). *Agent-based and individual-based modeling: A practical introduction.* Princeton: Princeton University Press). [↑](#footnote-ref-1)
2. The model described here is based on and similar to Sattenspiel, L. (2019, April 15). “St. Anthony flu” (Version 1.0.0). *CoMSES Computational Model Library.*  Retrieved from https://www.comses.net/codebases/758c8616-0d8a-44d4-a3fa-921f902b9561/releases/1.0.0/ [↑](#footnote-ref-2)
